# Tislelizumab immunotherapy combined with chemotherapy in the treatment of a patient with primary anterior mediastinal undifferentiated pleomorphic sarcoma with high PD-L1 expression: A case report and literature review

**DOI:** 10.3389/fonc.2023.1110997

**Published:** 2023-04-05

**Authors:** Hujuan Yang, Zhiquan Qin, Xianglei He, Qian Xue, Hongying Zhou, Jie Sun, Xiaoyi Li, Tongwei Zhao

**Affiliations:** ^1^ Graduate School of Clinical Medicine, Bengbu Medical College, Bengbu, Anhui, China; ^2^ Cancer Center, Department of Medical Oncology, Zhejiang Provincial People’s Hospital (Affiliated People’s Hospital, Hangzhou Medical College), Hangzhou, Zhejiang, China; ^3^ Cancer Center, Department of Pathology, Zhejiang Provincial People’s Hospital (Affiliated People’s Hospital, Hangzhou Medical College), Hangzhou, Zhejiang, China

**Keywords:** mediastinal tumor, undifferentiated pleomorphic sarcoma, immune checkpoint inhibitors, immunotherapy, tislelizumab

## Abstract

Undifferentiated pleomorphic sarcoma (UPS) is a rare and aggressive soft tissue tumor with a high degree of malignancy and rapid progression, usually occurring in the extremities, retroperitoneum, and abdomen, whereas it rarely arises in the mediastinum, and is treated mainly by surgical resection. The prognosis of patients with advanced sarcoma is poor, and doxorubicin monotherapy is the standard first-line chemotherapy for most advanced soft tissue sarcomas (STS), but the prognosis is generally unsatisfactory. Immune checkpoint inhibitors (ICIs) have been established as therapies for many solid cancers in recent years; however, evidence on the efficacy of ICIs in undifferentiated sarcoma is scarce, mostly consisting of small studies, and no ICIs are currently approved for use in sarcomas. We report a case of a middle-aged man with primary mediastinal UPS with high PD-L1 expression (TPS was approximately 80%) and TLS positive. The patient was treated with sequential tislelizumab monotherapy maintenance after 6 cycles of tislelizumab combined with epirubicin, efficacy evaluation was partial remission (PR), progression-free survival (PFS) was 8.5 months, and grade 1 fatigue was identified as an adverse event.

## Introduction

Soft tissue sarcomas (STSs) are tumors originating from the connective tissue stroma and account for approximately 1% of all cancers (1). Primary mediastinal sarcomas are relatively rare and aggressive soft tissue tumors, accounting for 10% of primary mediastinal tumors and 1% of all STSs (2). Because of its rarity and the high heterogeneity in the natural course of the disease, STS was less frequently described in previous reports, and only small retrospective case series or reports have been published, with studies revealing median overall survival (OS) of 27.2 months (3). Undifferentiated pleomorphic sarcoma (UPS) represents a group of heterogeneous soft tissue tumors with no clear differentiation direction, consisting of multiple histologically undifferentiated atypical cells, previously known as malignant fibrous histiocytomas (MFH), reclassified as unclassifiable/undifferentiated sarcoma (US) in the fourth edition of the World Health Organization (WHO) classification released in 2013. UPS usually occurs in the extremities, retroperitoneum, and abdomen (3, 4), and it rarely arises in the mediastinum and thorax. The main factors affecting the prognosis are the presence of tumor infiltration and metastasis and the completeness of surgery (3, 5).

The prognosis of patients with advanced sarcoma is poor, and doxorubicin monotherapy has been the standard first-line chemotherapy for most advanced STSs. However, the prognosis is generally unsatisfactory, thus there is an urgent need to develop new effective therapies. In recent years, targeted therapies using immune checkpoint inhibitors (ICIs) to block the binding between programmed cell death-1 (PD-1) and programmed cell death ligand-1 (PD-L1) achieved good responses in various malignancies such as lung cancer (6) and melanoma (7). Monoclonal antibodies against PD-1 and PD-L1 can activate T lymphocytes by blocking the PD-1/PD-L1 signaling pathway, thereby preventing tumors from achieving immune evasion (8). Treatment response is often associated with the presence of tumor-infiltrating lymphocytes (TILs) and PD-L1 expression in tumor and immune cells (9). The limited activity of PD-1 inhibitors in selected STS that may be explained by an immunosuppressive tumor microenvironment resulting from macrophage infiltration and IDO1 (inhibitory enzyme indoleamine 2,3-dioxygenase 1) pathway activation (10). Evidence on the efficacy of ICIs in UPS is still scarce and mostly small-sample studies, such as the phase II study (SARC028) which showed certain efficacy of UPS treated with ICIs (11), so there is no officially approved ICIs for the treatment of sarcomas.

In this study, we report a patient with primary UPS with high PD-L1 expression (tumor proportion score [TPS] = 80%) originating from the anterior mediastinum, who was treated with sequential tislelizumab monotherapy maintenance after 6 cycles of tislelizumab immunotherapy combined with epirubicin chemotherapy, the patient responded well, and no obvious side effects was observed. A partial response (PR) was achieved, and progression-free survival (PFS) was 8.5 months.

## Case description

In June 2021, a 61-year-old man patient presented to a local hospital with chest pain, who had no obvious medical history and denied a family history of tumor. Computed tomography (CT) of the chest showed a square cardiac mass considered potentially malignant. Superficial ultrasound of the neck revealed abnormal lymph nodes in the IV region of the left side of the neck near the supraclavicular region, with metastatic foci considered. The immunohistochemical (IHC) staining results were as follows: Ki67 index, 80%; EMA, focal positivity; CD20 lymphocyte positivity; CD3 lymphocyte positivity; CD34, no tumor thrombus; LNL-1 positivity (no deletion); and negativity for Cam5.2, S-100, HMB45, mclan-A, and MLM-1. Mediastinal puncture was performed in a local hospital, and the pathological results showed: First, a large number of tumor cells were diffusely distributed among the fibrous connective tissues, with round cells, translucent or eosinophilic cytoplasm, and round or ovoid nuclei, and some nucleoli were visible, potentially indicating thymic malignancy. Second, the left subclavian lymph node puncture specimen revealed a malignant tumor with lymph node metastasis. US with lymph node metastasis was considered according to the aforementioned results combined with IHC data. The patient’s symptoms did not improve after symptomatic treatment in the local hospital.

**Figure 1:**
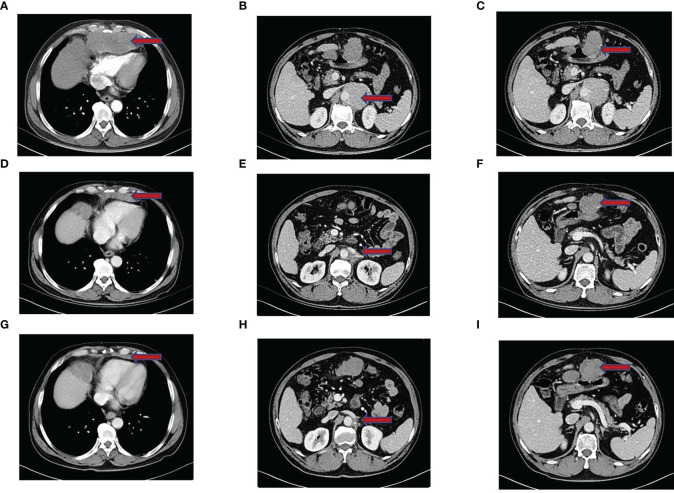
Imaging performance. **(A–C)** Enhanced CT of the chest and abdomen before treatment showed a dense mass shadow of soft tissue in the anterior mediastinum, multiple retroperitoneal lymph nodes enlarged and fused into a mass, and left liver occupancy. **(D–F)** After 2 months of tislelizumab combined with epirubicin treatment, the CT showed a significantly smaller lesion shadow than before, and the efficacy evaluation was PR. **(G–I)** In April 2022, Chest CT showed that the size of the anterior mediastinal hypodense foci was similar to that before. CT of abdominopelvic cavity showed multiple posterior peritoneal and perihepatic occupations with increased lesion extent compared to previous CT, and some of the lesions were new. Suspicious new nodules in segment VI of the liver, and efficacy evaluation of PD.

**Figure 2:**
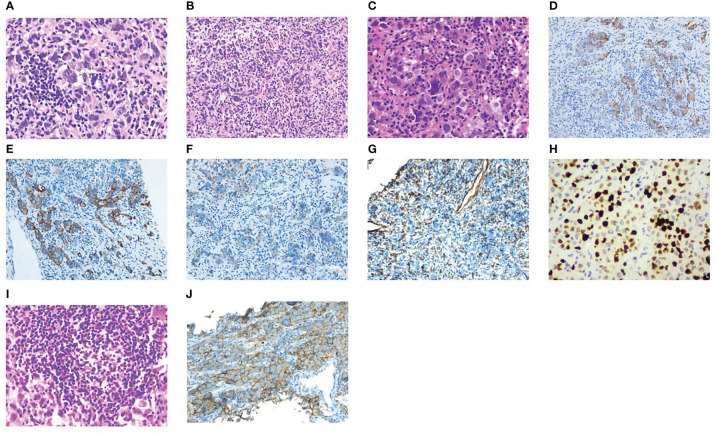
Pathological results. **(A–C)** Structural destruction in lymphoid tissue, diffuse hyperplasia of heteromorphic tumor cells, large size of tumor cells, obvious pleomorphism, spindle-shaped and polygonal, large and deep stained nuclei and irregular pseudoinclusion bodies in nuclei, multinucleated and giant tumor cells in some tumor cells, multiple nucleoli, mitotic images are easy to see (magnification × 40, 20, 40). **(D–H)** IHC staining showed sequentially CKpan, CAM5.2, EMA, Vimentin, Ki67 (+). **(I)** Localized aggregation of lymphocytes to form nodules, and the formation of a central germinal center of the nodule is seen, consistent with tertiary lymphoid structures. **(J)** Positive for PD-L1 (Dako22C3).

Then, he was transferred to our hospital for treatment on July 30, 2021. After admission, only alpha-fetoprotein AFP (53.4 ug/l) and neuroenolase (20.7 ng/l) levels exceeded normal values, whereas the levels of other tumor markers such as carcinoembryonic antigen (CEA), cytokeratin (CYFRA), and precursor gastrin-releasing peptide (ProGRP) were normal. Chest CT showed mediastinal lymphadenopathy and enhancement, a soft-tissue density mass and nodular shadow in the anterior mediastinum with a maximum diameter of 100 mm; involvement of the anterior chest wall, pericardium, and liver capsule; retroperitoneal lymphadenopathy merged into the mass; and left hepatic occupancy on August 2, 2021 (**Figure 1A**). Enhanced abdominal and pelvic CT on August 3, 2021 uncovered multiple masses in the posterior peritoneum, anterior gastric margin, perihepatic and anterior mediastinum, which were considered malignant lesions (**Figures 1B, C**). Diagnostic ultrasound-guided percutaneous lymph node aspiration biopsy revealed five gray-white puncture tissues, 1.0*2.2cm in length and 0.1cm in diameter. Tertiary lymphoid structures (TLS) were seen in the metastatic cancer tissue of the cervical lymph nodes, and the pathological findings suggested French Federation of Cancer Centers Sarcoma Group (FNCLCC) grade III. IHC staining showed negativity for vimentin (+), CD99 (weak +), CD163, CD1a, CD68, CD5/6, CD21, CD23, P63, CD35/CR1, CD79a, PAX-5, D2-40, and WT-1 (**Figure 2**). Combined with the previous IHC results, the findings were consistent with a diagnosis of UPS. PD-L1 was highly expressed, and the TPS was approximately 80% (Dako22C3).

**Figure 3:**
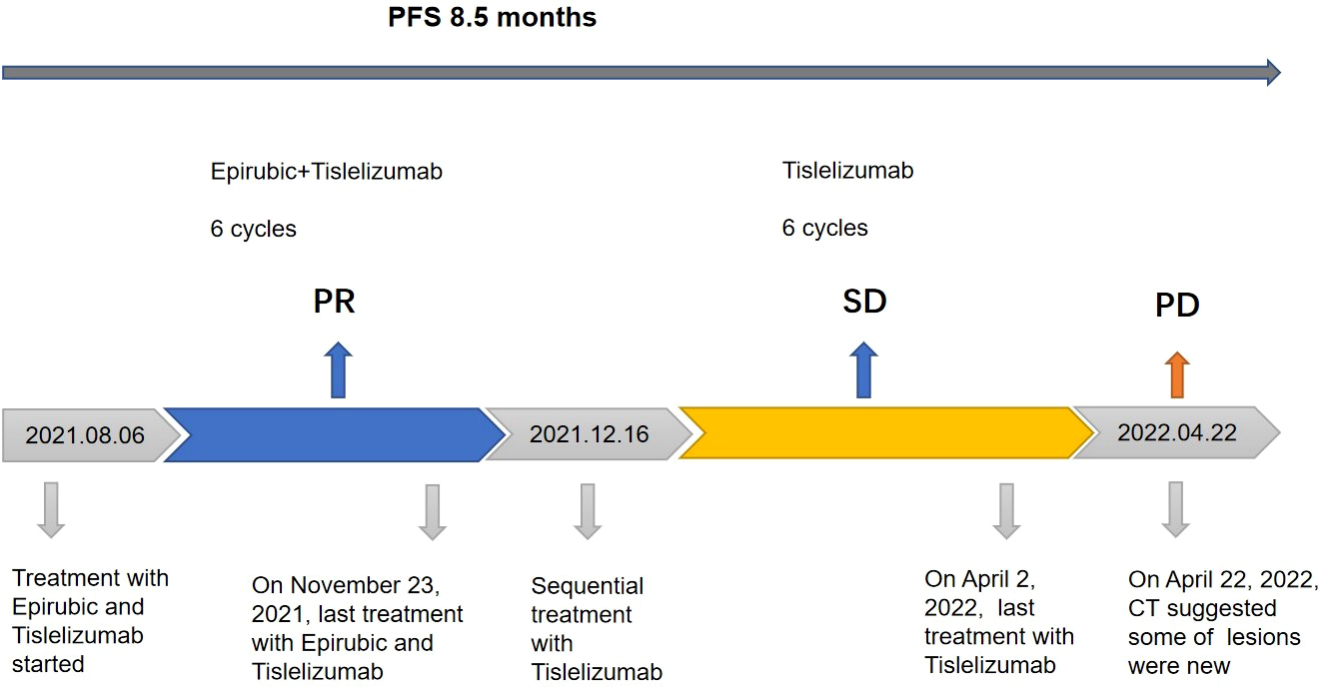
Review of treatment process. Treatment process and effect evaluation of the patient. PFS, progression-free survival; PR, partial response; SD, stable disease; PD, progressive disease.

According to the American Joint Committee on Cancer (AJCC) 8th edition staging, the patient was cT3N1M1 (stage IV) and invaded the pericardium, so the patient lost the opportunity for surgery. From August 6, 2021 to November 23, 2021, the patient was treated with epirubicin 130 mg combined with tislelizumab 200 mg Q3W for 6 cycles. Two months later, CT was repeated, and the lesion had significantly regressed (**Figures 1D–F**). According to the Response Evaluation Criteria in Solid Tumors (RECIST) criteria, the efficacy was evaluated as PR. During chemotherapy, the patient developed grade I fatigue, and other adverse reactions were not obvious. The patient was readmitted to the hospital for treatment for occasional chest tightness and shortness of breath, and cardiac ultrasound revealed an ejection fraction of 61% on December 16, 2021. Considering that the decrease in cardiac function was possibly related to cardiotoxicity caused by epirubicin, meanwhile, epirubicin has been treated with for 6 cycles, therefore we discontinued epirubicin and sequentially treated with tislelizumab monotherapy for maintenance. In April 2022, repeat chest CT suggested a stable disease (**Figure 1G**); however, abdominal CT uncovered multiple lesions in the posterior peritoneal, perigastric, and perihepatic occupancies with an increased range of lesions compared to the previous findings. Some lesions were new, suspicious nodules in segment VI of the liver, newer than before, and metastases to be excluded (**Figures 1H, I**). Evaluating progression, the patient achieved PFS of 8.5 months, which exceeded the average survival time of previously reported cases. After progression, the patient was treated with gemcitabine combined with tislelizumab and was switched to anlotinib monotherapy due to the development of interstitial pneumonia. The efficacy evaluation was SD and PD, respectively. PFS was 2.8 months and 2.3 months, respectively. On January 28, 2023, the patient eventually died of obstructive jaundice due to intrahepatic and extrahepatic bile duct obstruction caused by tumor progression. The primary treatment timeline is shown in **Figure 3**.

## Discussion

Primary mediastinal tumors are uncommon, predominantly occur between 30 and 40 years of age, although they can occur at any age. Symptoms vary depending on the anatomical location, size, and histology of the tumor. They are usually asymptomatic and first detected on a routine chest radiograph. The most common symptoms include chest pain, abdominal pain, cough, dyspnea, and masses (12, 13). The present patient presented with chest pain as the first symptom. If a mediastinal mass is suspected, CT is the standard imaging modality to clarify the nature, density, and relationship to adjacent tissues of the mass, and magnetic resonance imaging (MRI) is helpful for identifying possible vascular invasion or spinal cord involvement (12). Because UPS lacks special clinical manifestations in the early stage and features no specific changes on auxliary examinations, histopathological examination has become the gold standard for its diagnosis, presenting microscopically as a high-grade sarcoma without any specific differentiation direction. According to the majority of publications, UPS lacks specific immunohistochemical markers, which is an excluded diagnosis (14). Vimentin, WT-1, CD99, and EMA can be expressed in UPS (14, 15). However, Masamichi (16) et al. found that UPS only exhibited diffuse and strong cytoplasmic positivity for vimentin. Furthermore, in a clinicopathological and immunohistochemical study of 46 germ cell tumors with sarcoma components, 4 cases of US were only positive for vimentin (17). Therefore, vimentin positivity can improve the diagnostic specificity during the diagnostic process. Therefore, Vimentin (+) can improve the specificity of the diagnosis in the diagnostic process.

Because of the high malignancy, early metastasis, rapid progression, and poor prognosis of UPS, surgery is currently the main treatment method. The main factors affecting the prognosis of UPS are the FNCLCC grading (tumor differentiation, mitotic count, tumor necrosis and histological grade), tumor size and depth of infiltration, and the R0 surgery (3, 5, 18), our patient’s FNCLCC grade III suggests poor prognosis. According to the 8th edition of AJCC classification, the patient in this article had T3N1M1 stage IV and the tumor had invaded the pericardium, so the patient was deprived of surgery and opted for pharmaceutical anti-tumor therapy. In recent years, the treatments for advanced sarcoma have included chemotherapy and targeted drugs. Common chemotherapy drugs include anthracyclines, ifosfamide, and dacarbazine (19). Anthracycline-based chemotherapy is the standard first-line treatment, and its side effects include bone marrow suppression and cardiotoxicity (20). Epirubicin, a common anthracycline, is less cardiotoxic than conventional chemotherapeutic drugs (doxorubicin) (21), and a retrospective analysis illustrated that compared with patients who did not receive adjuvant chemotherapy, patients treated with epirubicin-based obtained more obvious benefit (22), so the NCCN clinical practice guidelines also recommend epirubicin for first-line chemotherapy in sarcoma. Targeted therapy is a powerful way to improve the efficacy of current standard chemotherapy in STS, especially CDKIs and TKIs targeting CDK4/6 and MDM2 (23, 24). In a placebo-controlled phase 3 trial, pazopanib demonstrated better outcomes in patients with advanced STS who failed standard chemotherapy, with significantly longer mPFS (4.6 months vs. 1.6 months) and OS (12.5 vs. 10.7 months) in the pazopanib group (PALETTE) (25). In addition, a prospective study of advanced STS in children and adults showed that the addition of pazopanib to preoperative radiotherapy improved pathological remission rates compared with radiotherapy alone (ARST1321) (26). These results suggest that targeted therapy is an effective option in the post-treatment of STS. This patient was newly diagnosed and the standard treatment was chemotherapy, so we chose epirubicin with lower cardiotoxicity. Nevertheless, the patient still experienced associated cardiotoxicity during epirubicin treatment and a substantially decreased ejection fraction, so we discontinued epirubicin and switched to tislelizumab for maintenance treatment.

So far, there is no guideline recommending immunotherapy for advanced sarcoma, mostly in case reports and small series. We reviewed previous published studies of ICIs for treatment of UPS (**Table 1**), which illustrated that immune checkpoint blockade can induce durable responses, and PD-1 inhibitors have certain anti-tumor activity for advanced UPS, patients with positive PD-L1 expression showed better therapeutic expression (11, 27), this is consistent with the case we have reported. In addition, there are two ongoing clinical studies of PD-1 combination regimens for the treatment of advanced STS with ICIs. One clinical trial (https://clinicaltrials.gov/ct2/show/NCT04420975) enrolling patients with resectable STS investigates preoperative give nivolumab joint BO - 112 immune therapy effect, The first safety data from the combination therapy in patients receiving preoperative radiotherapy before surgical excision is expected in early 2024. Another phase II randomized study (https://clinicaltrials.gov/ct2/show/NCT04480502) designed to examine the safety and efficacy of envafolimab in combination with or without ipilimumab in patients with UPS who progressed after chemotherapy (ENVASARC), preliminary data from the first 20 enrolled patients showed that envafolimab was well tolerated when treated alone and in combination with ipilimumab.

**Table 1:** Previous reports of UPS treated with ICIs.

Author	Report type	number	Regimen	Efficacy	Predictive biomarkers
Toulmonde, M., etc. (10)	Clinical Trial	16	Pembrolizumab and Cyclophosphamide	The median PFS for 1.4 months.	PD-L1 expression was 64% and IDO expression was 29% in the UPS
Tawbi, H.A., etc. (11)	Clinical Trial	10	Pembrolizumab	The median PFS for 30 weeks. The ORR of UPS was 40%.	Three patients (all from UPS) were determined to be positive for PD-L1 (TPS> 1%)
Livingston, M.B., etc. (27)	Clinical Trial	4	Pembrolizumab and Doxorubicin	The ORR of UPS was 100%.	The evaluable patients had PD-L1 H-Score ≥5% had a greater ORR (63.6%) than those with an H-score of <5% (22.2%)
Spalato-Ceruso, M., etc. (28)	Clinical Trial	1	Pembrolizumab and Cyclophosphamide	The ORR of UPS was 100%. (one patient was partial respons).	high circulating soluble pro- grammed death-1 ligand (sPD-L1) was the sole protein significantly associated with worse PFS and OS.
D’Angelo, S. P., etc. (29)	Clinical Trial	10	Nivolumab and bempegaldesleukin	The median PFS for 2.4 months. The ORR of UPS was 20%(2/10).	Increased numbers of T cells and elevated PD-1 expression were associated with improved ORR and prolonged PFS in the UPS.
Somaiah, N., etc. (30)	Clinical Trial	5	Tremelimumab and durvalumab	The ORR of UPS was 20%(1/5).	At baseline, one (33%) of three responders were PD-L1-positive.
Cheung, L. S., etc. (31)	Case report	2	Case1: Anti-PD-1 treatment. Case2: Anti-PD-1 and anti-CTLA-4 combined immunotherapy	Case1: No metastasis for 16 months. Case2: Disease was stable at 18 months.	Both patients had high levels of PD-L1 expression. TMB was 33 and 43 mutations/MB, respectively.
Li, Y., etc. (32)	Case report	1	toripalimab and anlotinib	23 months PFS.	PD-L1 expression in tumor was 90%.
Arora, S., etc. (33)	Case report	1	Pembrolizumab and pazopanib	DOR>10months.	PD-L1 expression was 25%.
Present case	Case report	1	Tislelizumab and epirubicin	8.5 months PFS.	The PD-L1 expression was approximately 80%. Presence of TLS.

PFS, Progress free survival; ORR, Objective response rate; DOR, Duration of response.

The interaction between tumor immune microenvironment and immunotherapeutic response is an ongoing area of research. Currently, the biomarkers predicting ICIs for STS treatment are still unclear, with hotspots of research including PD-L1 expression, IDO1, and TLS, etc. (**Table 1**). The role and mechanism of PD-L1 expression in STS remain unclear, but studies indicated that increased PD-1 expression on TILs and increased PD-L1 expression on STS cells predict poor prognosis (34, 35), suggesting that in advanced STS, PD1/PD-L1 blockade may increase the anti-tumor activity of tumors with high PD-L1 expression by reactivating suppressed T cells. Previous studies have shown that patients with higher PD-L1 expression have better efficacy than patients with lower expression in STS after anti-PD-1 or anti-PD-L1 therapy (11, 27). Partial remission was observed in a phase II clinical trial of pembrolizumab in combination with cyclophosphamide in PD-L1 positive patients with mild IDO1-positive immune cells in their tumors (10). To date, several pieces of evidence suggest that the efficacy of anti-PD1/PD-L1 in sarcomas is associated with the presence of TLS (28, 36, 37), and they mentioned that IHC found positivity of CD20 and CD3 can reflect the presence of TLS. In a phase II study (PEMBROSARC) (36), pembrolizumab combined with low-dose cyclophosphamide in patients with STS enrolled a cohort was selected based on the presence of TLS (n = 30). The 6-month non-progression rate (NPR) was 40% and the median PFS was 4.1 months, suggesting that TLS may also serve as a predictive biomarker. The patient in this report had high PD-L1 expression, TLS visible in cancer tissue, and positive CD20 and CD3, immunotherapy may have high efficiency. After full communication with the patient and his family, they agreed to use off-label tirelizumab combined with epirubicin regimen for PFS reached 8.5 months, showing good efficacy. In addition, a number of clinical studies currently underway have demonstrated predictive biological markers relevant to immunotherapy. For example, in the CAIRE trial (https://clinicaltrials.gov/ct2/show/NCT04705818) and the PEMBROCABOSARC trial (https://clinicaltrials.gov/ct2/show/NCT05182164), respectively studying TLS, blood lymphocyte levels and tumor immune cell levels may be reliable predictive biomarkers of STS treated by ICIs.

STS is generally described as having low PD-L1 expression with response rates range 5%-18% (11, 38). Sarcomas with PD-L1 expression include angiosarcoma, chondrosarcoma, Ewing’s sarcoma, rhabdomyosarcoma, synovial sarcoma, and UPS, among which high PD-L1 expression tends to be more common in UPS (8, 34). Zhang et al. (8), used the SP263 assay to measure PD-L1 expression in chondrosarcoma, liposarcoma, and UPS, respectively, and observed increased response rates in the UPS group, with TPS >50% observed in 8 tumors (8/96). To our knowledge, there are very few reports of PD-1 inhibitors for UPS PD-L1 high expression. In this report, our patient is a middle-aged man with primary mediastinal UPS, with PD-L1 TPS reaching 80% of tumor cells and TLS visible in pathological sections. After tislelizumab immunization combined with epirubicin chemotherapy, the patient’s lesions regressed significantly. The efficacy evaluation was PR, and PFS was 8.5 months with tolerable adverse effects. OS was 17.5 months.

Conventional first-line epirubicin and doxorubicin chemotherapy has a PFS of 3-4 months (39), and the patient in this case had a PFS of 8.5 months, which is about twice the PFS of conventional chemotherapy. Our reported results show that epirubicin combined with tislelizumab is more effective than conventional first-line chemotherapy in UPS with high PD-L1 expression, and high PD-L1 expression and TLS may be effective biomarkers to predict the effectiveness of anti–PD-1 therapy. Early detection of PD-L1 expression and application of anti–PD-1/PD-L1 antibodies may be a promising strategy for the treatment of UPS patients with high PD-L1 expression. Of course, there are some limitations in this paper. Firstly, only one patient was reported in this paper, and the data are limited. In the future, we need more large-scale randomized controlled trials to study the efficacy and safety of ICIs in mediastinal UPS patients. Secondly, the mechanism of immunotherapy for UPS is not proposed in this paper, and corresponding basic research is needed to explore the potential mechanism of immunotherapy in the future.

## Data availability statement

The original contributions presented in the study are included in the article/supplementary material. Further inquiries can be directed to the corresponding author.

## Ethics statement

Written informed consent was obtained from the individual(s) for the publication of any potentially identifiable images or data included in this article.

## Author contributions

HY searched literature, wrote the original draft, revised and submitted the manuscript. HY, TZ, and XH retrieved and analyzed patient data and imaging. TZ and ZQ reviewed and edited the manuscript. QX and HZ made substantial contributions in data retrieval and data interpretation. JS and XL planned the case report. All authors contributed to the article and approved the submitted version.
